# Fractionation statistics

**DOI:** 10.1186/1471-2105-12-S9-S5

**Published:** 2011-10-05

**Authors:** Baoyong Wang, Chunfang Zheng, David Sankoff

**Affiliations:** 1 Department of Mathematics and Statistics, University of Ottawa Ottawa, Canada K1N 6N5

## Abstract

**Background:**

Paralog reduction, the loss of duplicate genes after whole genome duplication (WGD) is a pervasive process. Whether this loss proceeds gene by gene or through deletion of multi-gene DNA segments is controversial, as is the question of fractionation bias, namely whether one homeologous chromosome is more vulnerable to gene deletion than the other.

**Results:**

As a null hypothesis, we first assume deletion events, on one homeolog only, excise a geometrically distributed number of genes with unknown mean *µ*, and these events combine to produce deleted runs of length l, distributed approximately as a negative binomial with unknown parameter *r*, itself a random variable with distribution *π*(·). A more realistic model requires deletion events on both homeologs distributed as a truncated geometric. We simulate the distribution of run lengths *l* in both models, as well as the underlying *π*(*r*), as a function of *µ*, and show how sampling *l* allows us to estimate *µ*. We apply this to data on a total of 15 genomes descended from 6 distinct WGD events and show how to correct the bias towards shorter runs caused by genome rearrangements. Because of the difficulty in deriving *π*(·) analytically, we develop a deterministic recurrence to calculate each *π*(*r*) as a function of *µ* and the proportion of unreduced paralog pairs.

**Conclusions:**

The parameter *µ* can be estimated based on run lengths of single-copy regions. Estimates of *µ* in real data do not exclude the possibility that duplicate gene deletion is largely gene by gene, although it may sometimes involve longer segments.

## Background

Whole genome doubling (WGD) triggers the wholesale shedding of duplicate genes through processes such as epigenetic silencing, pseudogenization, and deletion of chromosomal segments containing one or more genes [1-5]. The extent to which this *paralog reduction* is a gene-by-gene *inactivation* process [6] targeting redundant copies at random points throughout the genome, or a consequence of largely random excision, elimination of excess DNA [2], is controversial and likely varies from one phylogenetic domain to another. The distinction between these two processes is not sharp: The inactivation effect may be produced not only by pseudogenization and various suppression and silencing mechanisms but also by the actual excision of a small but critical region of a gene or promoter. Conversely, the apparent excision of two or more adjacent genes may rather be due to any of a variety of genetic, epigenetic or functional interactions, rather than the deletion of a DNA fragment. Nevertheless, the determination of whether paralog reduction is a gene-by-gene process or the deletion of longer stretches DNA is key to understanding the dynamics of genome evolution, not only following WGD, but as part of the continual innovative expansion and simplifying shrinkage of genomes over time.

The other face of paralog reduction is the process of fractionation. When a duplicate gene is lost, it may be lost from one copy (*homeolog*) of a chromosome or the other. When compared to the pre-WGD genome, or to a closely related but unduplicated genome, this creates an interleaving pattern, such that it is only by *consolidating*[4] the two homeologous single-copy regions that the full original gene complement becomes apparent. That the consolidated region is directly comparable to homologous regions in related genomes is due to the fact that single-copy genes are rarely deleted - of the two duplicates created by WGD, it is unlikely that both are deleted, for obvious functional reasons.

Fractionation is an important evolutionary process whenever WGD occurs, and is of particular interest for comparative genomics, since it results in a genome that is highly scrambled with respect to its pre-WGD ancestor. The study of fractionation also brings up the question of *bias*: Are paralogs always or generally lost from the same “side”, or are they lost randomly from one homeologous chromosome or the other [3-5,7]?

In this paper, we analyze paralog reduction and fractionation in terms of two models, one easier to analyze but the other more realistic. First we model paralog reduction on only one of the two homeologous chromosomes as a series of excisions of geometrically distributed lengths and show how to use the observed run lengths of single-copy genes on the other chromosome to estimate the parameter of the deletion length distribution.

In the second model we allow excisions on both homeologous chromosomes, and model deletion lengths in terms of truncated geometric distributions to account for the above-mentioned prohibition against deleting single-copy genes.

This work is essentially the creation of a simple, one-parameter “null” model of paralog reduction, where deletion is by random events involving geometrically distributed (with mean *µ*) numbers of genes on one homeologous chromosome or randomly on both of them. This sets up the possibility of statistical tests of real WGD descendants, to see if the geometric hypothesis is acceptable and to see if fractionation is unbiased or not. We will not explicitly investigate the alternative hypotheses of gene-by-gene excision or biased fractionation; our task here, aside from estimating the parameters of our model, is simply to set up the null statistical model with a view to eventually developing useful statistical tests of hypothesis for this problem.

In a previous study of post-WGD evolution [3], we took chromosomal rearrangement events into account. In the present paper, we do not incorporate rearrangement into our model, but we do reanalyze some of the data, to explore the effects of genome rearrangement processes in confounding the evidence of fractionation and to suggest a way of redressing the loss of information.

The lengths of runs of undeleted genes may be considered independent samples from a geometric distribution, and the lengths of runs of deleted genes are also independent, but we show that the deletion events making up a run of deleted genes are not independent. As a consequence, the distribution of deleted run lengths seems beyond the scope of straightforward mathematical derivation. The major analytical and computational result of this paper is the construction, implementation and evaluation of a deterministic recurrence to calculate the distribution of the number of deletion events per run as a function of *µ* and the proportion *θ* of unreduced paralog pairs.

## The models

### The structure of the data

The data on paralog reduction are of the form (*G*, *H*), where *G* and *H* are binary sequences indexed by ℤ, satisfying the condition that *g*(*i*) + *h*(*i*) > 0. This condition models the prohibition against deleting both copies of a duplicated gene. We may also assume that whatever process generated the 0s and 1s is homogeneous on ℤ.

The sequence *G* + *H* consists of alternating runs of 1s and 2s. We denote by *p*(*l*)*, l* ≥ 1 the probability distribution of length of runs of 1s. For any finite interval of ℤ we denote by *f*(*l*), *l* ≥ 1 the empirical frequency distribution of length of runs of 1s.

The use of ℤ instead of a finite interval is consistent with our goal of getting to the mathematical essence of the process, without any complicating parameters such as interval length. In practice, we will use long intervals of 100,000 or 300,000 so that any edge effects will be negligible. See [3] and the section below on 15 WGD-descendant genomes for *ad hoc* ways of handling biological scale intervals.

### One-sided deletion

In this case *h*(*i*) = 1, for *–∞ < i < ∞*. We assume a continuous time process, parameter λ(*t*) > 0, only to ensure no two events occur at the same time. We start (*t* = 0) with *g*(*i*) = 1 for all *i*. At any *t* > 0, consider any *i* where *g*(*i*) = 1. With probability *λ*(*t*)*dt*, the following *deletion event* occurs, *anchored* at position *i:* we choose a positive number *a* according to a geometric variable **y** with parameter 1*/µ*, i.e.:(1)

and we convert *g*(*i*) = 0, *g*(*i* + 1) = 0, … , *g*(*i* + *a –* 1) = 0, unless one or more of these is already 0, in which case we skip over it and continue to convert the next available 1s into 0s, until a total of *a* 1s have been converted. This is a natural way to model the excision process, since deletion of duplicates and the subsequent rejoining of the DNA directly before and directly after the excised fragment means that this fragment is no longer “visible” to the deletion process. Observationally, however, we know deletion has occurred because we have access to the sequence *H*, which retains copies of the deleted terms.

When the deletion event has to skip over previous 0s, this hides the anchor *i* and length *a* of previous deletion events. Denote by **r** the random variable indicating the total number of deletion events responsible for a run. Then, given **r** = *r*, the run length **z** is distributed as the sum of *r* geometric variables, which would result in the negative binomial distribution:(2)

if these geometric variables were independent. As we shall see later, however, the hypothesis of independence does not hold.

If we observe *G* at some point in time, as in the last row of Table 1, all we can observe are the run lengths of 0s and 1s. We cannot observe the *a, i* or *r*, while *t* and λ(*t*) are unknown and, as we shall see, only mathematical conveniences that do not enter into our calculations. The parameter about which we wish to make statistical inferences is the deletion length distribution parameter *µ*, since it is this quantity that is at the heart of the biological controversy about paralog reduction. This inference therefore can only be based on the run lengths and the proportion of remaining 1s. If the probability distribution of **r** is *π*(·), the distribution of run length **x** is approximately:(3)

**Table 1:** One-sided model

event	*i*	*a*	-7	-6	-5	-4	-3	-2	-1	0	1	2	3	4	5	6	7	8	*r*
start			1	1	1	1	1	1	1	1	1	1	1	1	1	1	1	1	
1	-1	3	1	1	1	1	1	1	0	0	0	1	1	1	1	1	1	1	1
2	-4	1	1	1	1	0	1	1	0	0	0	1	1	1	1	1	1	1	1,1
3	4	4	1	1	1	0	1	1	0	0	0	1	1	0	0	0	0	1	1,1,1
4	-5	4	1	1	0	0	0	0	0	0	0	0	1	0	0	0	0	1	3,1

Four deletion events leading to two runs of 0s. Illustrates the creation of a long run with *r* = 3 subsuming two previous shorter runs. Note that *r* is not observable.

The one-sided model is an extreme version of biased fractionation and is not meant to model any real situation. It is, however, relatively tractable and hence provides a mathematical framework for understanding more realistic cases.

### Two-sided deletion

In a more realistic model, deletions can occur both in sequence *G* and sequence *H* as in Table 2. Thus before choosing a position *i*, we chose either *G* or *H* with probability *ø* and 1 – *ø*, respectively. The default we shall study here, , represents unbiased fractionation. Then position *i*, where *g*(*i*) + *h*(*i*) = 2 and geometric variable *a* are chosen as before.

Suppose *G* is the chosen sequence. Then *g*(*i*) is set to 0, *g*(*i* + 1) is set to 0, and so on until *g*(*a* + *i* – 1), unless we first reach a position *j* where *g*(*j*) is already 0, in which case we skip as before, or until we reach a position *k* where *h*(*k*) = 0. In this case, we cannot continue to delete, because *g*(*k*) is a single-copy gene, and we are prohibited from letting *g*(*k*) + *h*(*k*) = 0, for any *k*. In this case, we must truncate the geometric variable *a*, having already deleted only *k – i < a* terms.

**Table 2:** Two-sided model

event	*i*	*a*	-7	-6	-5	-4	-3	-2	-1	0	1	2	3	4	5	6	7	8	*r*
start			1	1	1	1	1	1	1	1	1	1	1	1	1	1	1	1	
			1	1	1	1	1	1	1	1	1	1	1	1	1	1	1	1	

1	-1	3	1	1	1	1	1	1	0	0	0	1	1	1	1	1	1	1	1
			1	1	1	1	1	1	1	1	1	1	1	1	1	1	1	1	

2			1	1	1	1	1	1	0	0	0	1	1	1	1	1	1	1	1,1
	-4	1	1	1	1	0	1	1	1	1	1	1	1	1	1	1	1	1	

3	4	4	1	1	1	1	1	1	0	0	0	1	1	0	0	0	0	1	1,1,1
			1	1	1	0	1	1	1	1	1	1	1	1	1	1	1	1	

4			1	1	1	1	1	1	0	0	0	1	1	0	0	0	0	1	3,1
	-5	4	1	1	0	0	0	0	1	1	1	1	1	1	1	1	1	1	

Four deletion events affecting two homeologous chromosomes, leading to two runs of single-copy genes. The fourth step illustrates how further deletion (at *i* = –1) and the “skip” process (at *i* = 2) are blocked when a single-copy gene is encountered (*i* = –1) on the homeologous chromosome, truncating the geometric variable *a*.

In this model, the deletion length is no longer geometric but a mixture of geometric and truncated geometric variables, and run length is no longer negative binomially distributed, even approximately.

## Results

### Simulations to determine *π*

**Figure 1:**
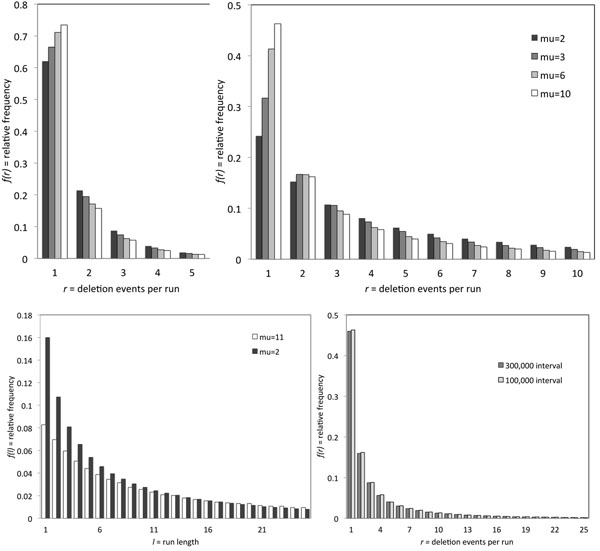
**Run statistics** Distribution of number of deletion events *r* composing each run when the proportion of sequence deleted is 0.5 (top left) and 0.9 (top right). Distribution of run length, reflecting a mixture of negative binomial distributions, for two values of the parameter of the underlying geometric distribution (bottom left). Identical results for simulation interval of 100,000 or 300,000 genes (bottom right).

We carried out a simulation of the one-sided model on an interval of ℤ of length 100,000. The top row of Fig. 1 compares *π*(*r*) when *θ* = 0.5 and *θ* = 0.1, for *µ* = 2, 3, 6, and 11. We can see that the number of deletion events contributing to a run is somewhat dependent on *µ* when half of the the sequence has been deleted, but is strongly dependent when 90 % has been deleted. In the bottom row, the graph on the left shows that run length *l* is distributed very differently for *µ* = 2 and *µ* = 11, when the proportion of the sequence deleted is exactly the same. This strongly suggests that observing the run length distribution and the overall proportion of deletions should allow us to infer *µ*.

Finally, the remaining graph in Fig. 1 shows that any edge effects in our simulation are negligible. Whether we work with *G* and *H* on an interval of ℤ of length 100,000 or, as in another simulation, length 300,000, gives virtually the same results.

**Figure 2:**
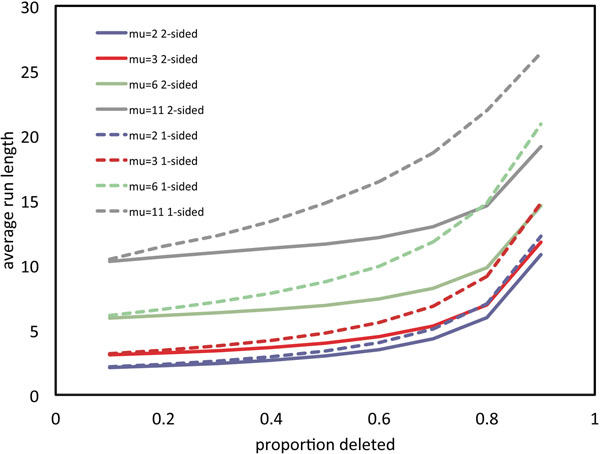
**Comparison of models** Average length of run of single copy genes in one-sided and two-sided models for *µ* = 2, 3, 6,11.

Figure 2 shows the relationship, in the one-sided and two-sided models, between the proportion of genes deleted, on one chromosome or the other, and the average run length, for a range of values of *µ*. This confirms our impression that average run length and overall proportion of deletion, both observable, can be used to infer *µ*.

### Non-independence of deletion events in a run

**Figure 3:**
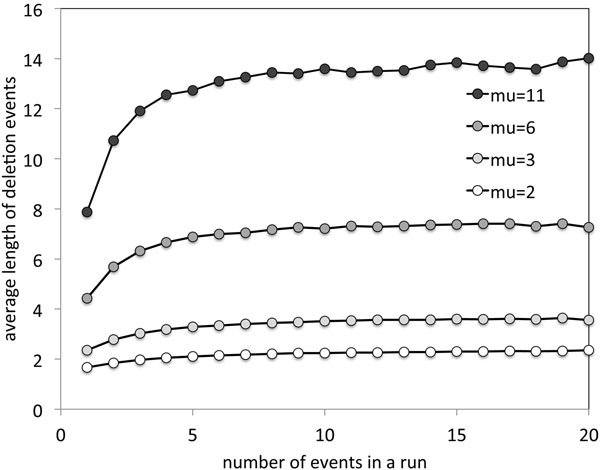
**Non-independence of deletion events in a run** Association of shorter deletions with smaller values of *r*.

A long deletion event within a run if undeleted genes has a greater chance of including all the following genes in that run, and possibly successive runs as well, than a short event deleting, say, only one or two genes. This implies that longer deletion events will tend to be grouped together in an event while short events are more likely to be in short runs. This the events making up a run are not chosen independently. This is reflected in the simulations in Fig. 3 for the case *θ* = 0.3.

## Application to 15 descendants of WGD events

To explore the relevance of our models for real genomes, we emphasize that we observe only the proportion *θ* of unreduced duplicates and the distribution of run lengths of single-copy genes on both homeologous chromosomes. (We can also observe the distribution of the run size of surviving paralog pairs, although models have not been developed for this.) We cannot observe *t* or λ. We cannot sample from the geometric distribution of deletion sizes, only their accumulation into runs, so that we cannot directly estimate its mean *µ*, the parameter of biological interest.

In [3], we studied 15 descendants of 6 ancient WGD events. In real genome sequences such as these, many or most runs of deleted paralogs will be impossible to identify; one or both of the homeologous regions will have been disrupted by inversions, translocations or other rearrangement events that juxtapose the surviving genes in the run with genes originally remote on the chromosome or from elsewhere in the genome.

**Figure 4:**
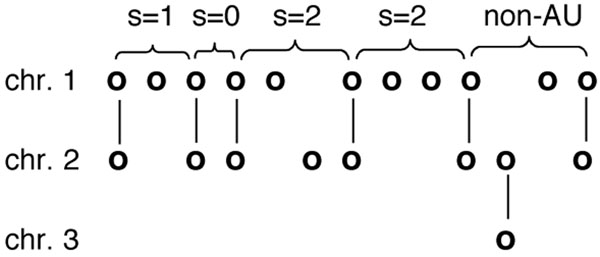
**Analytical units (AU)** The number of single copy genes *s* bounded by pairs of duplicates on chromosomes 1 and 2 is the sum of those on chromosome 1 and chromosome 2. The last two pairs of duplicates on chromosomes 1 and 2 do not border an AU because one of the genes between them has a paralog on chromosome 3. From [3].

We could, however, identify some two-sided undisrupted runs of single-copy genes, fractionated between two chromosomal regions. We searched for such *analytical units* (AU), two-sided runs flanked at either end by a pair of undeleted duplicate genes, with the two flanking genes on a chromosome having the same orientation, and including no intervening gene having a paralog somewhere outside the run, as in Fig. 4. It is statistically unlikely that such an AU configuration be produced by a series of compensating rearrangements, so that any rearrangements must have occurred entirely within the run, or have included the entire run intact plus the flanking duplicate pairs.

Among all the runs of single-copy genes in a WGD descendant genome, it is only the AU that can be used as evidence for the paralog reduction process, because it is only from these that we can reconstruct common conserved homeologous regions on two chromosomes (or remote regions on one chromosome).

Key characteristics of the genomes, their global properties and the properties of the two-sided runs are given in Table 3. *D = d/*(n – m) is the rearrangements per gene since WGD, where *d* is calculated only on the duplicates by the algorithm in [8], *n* is the total number of genes in the given genome and m is the number of single-copy genes.. The way *d* is calculated, there are between one and two breakpoints per rearrangement. We do not know how many rearrangements have affected the whole genome, duplicates and single-copy, but as a first approximation we assume that the probability that any adjacency will be disrupted by a rearrangement since the WGD is proportional to *D*, or *αD*. The proportionality constant *α ≤* 1 is unknown, but experience suggests  is a reasonable value.

**Table 3:** Data on 15 genomes

	*t*	*n*	*m*	1 – *θ*	
*S. cerevisiae*	150	5616	4498	0.89	6.0958
*C. glabrata*	150	5180	4382	0.92	5.3839
*V. polyspora*	150	5112	4164	0.9	4.922
*S. bayanus*	150	5857	4773	0.9	5.8297
*N. castelli*	150	5213	4053	0.88	5.0717

*Paramecium*	20	38626	14576	0.55	2.0299

populus	70	20082	7228	0.53	1.6402

*Arabidopsis*	50	25655	13267	0.68	3.6086

*fugu*	350	14251	12653	0.941	3.806
medaka	350	14564	13352	0.957	5.0629
stickleback	350	16726	14876	0.941	4.3792
*tetraodon*	350	17120	16088	0.969	6.876

chicken	450	10077	8495	0.915	3.6122
opossum	450	13339	11589	0.93	5.5507
human	450	13828	12144	0.935	3.818

Evolutionary inference about 15 descendants of 6 WGD events. *t*: time in My since event, *n*: total genes, *m*: single-copy genes, , *d*: halving distance [8],  average run length.

**Figure 5:**
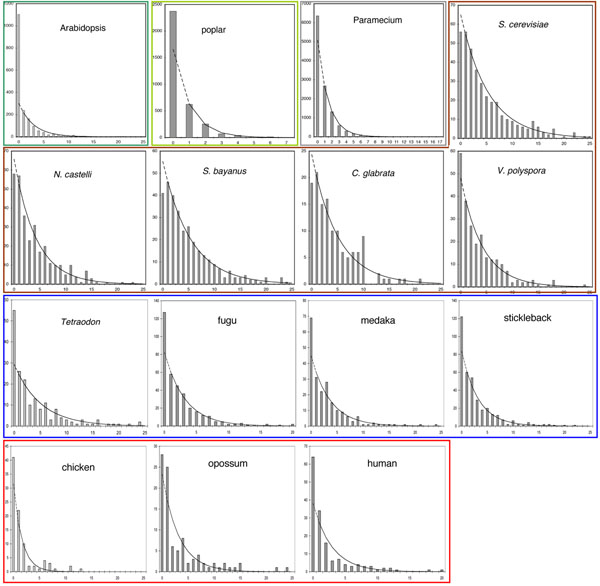
**Single copy runs in 15 WGD descendants** Distribution of length of run of single copy genes in 15 genomes descended from WGD events. Zero length indicates adjacent pairs of paralogs (i.e., not single-copy). Coloured boxes contain genomes descended from the same event. Frequencies of zero-length runs are not considered in the fitting by the geometric distributions shown. From [3].

In [3], the AU lengths in each of the 15 genomes were distributed as in Fig. 5. Since we model run length in terms of an unknown mixture of distributions involving *r* geometric or truncated geometric variables, where *π*(*r*) is unknown, we cannot infer *µ* directly. Nevertheless, we remark in the figure that the frequency distribution *f*(*u*) of the run lengths *u* ≥ 1 is closely approximated by a geometric distribution with mean  in all of the cases, except where there are few data. The mean  varies widely from genome to genome. In this section we will continue to make use of this approximation to help understand the data.

Consider an AU of length *u*. There are *u*+1 possible breakpoints in an AU of length *u*, including the two at either end of the run of single-copy genes involving the flanking duplicate genes, that could destroy the AU, according to definition.

Each adjacency in an AU will survive (not be disrupted by a breakage) an evolutionary period equal to the time from WGD with probability approximately (1 – *αD*). An AU of size *u* will survive with probability (1 – *αD*)^(*u*+1)^ Then *f*(*u*)/(1 – *αD*)^(*u*+1)^ is an estimate of the frequency of AUs of size *u* if there had been no rearrangements. The predicted relative frequency of run length becomes a geometric distribution with mean *ν*, where:(4)

Where , and(5)

**Figure 6:**
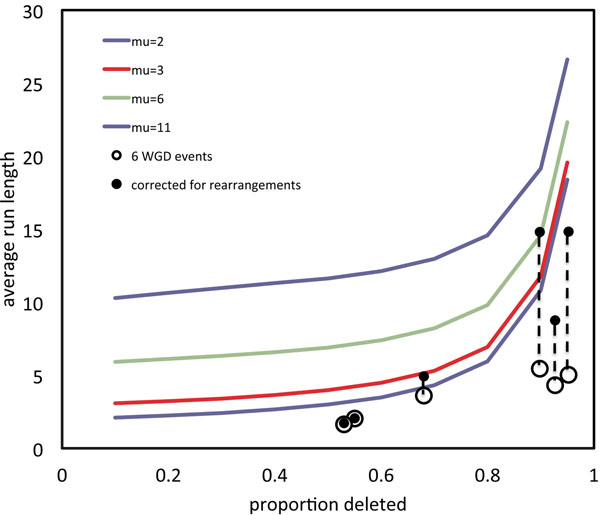
**Single copy runs in WGD descendants compared to model** Mean deletion run length in WGD descendants, uncorrected and corrected for rearrangements, compared to average length of run of single copy genes in the two-sided model for *µ* = 2, 3, 6 and 11.

Fig. 6 shows the two-sided curve of run length versus proportion deleted as in Fig. 2, but with the mean run length , averaged over the descendants of each of the six distinct WGD events superimposed. Each point is connected in the figure to the corrected mean *ν* calculated from Eq. 4. We used *α* = 0.5. This somewhat arbitrary choice is bounded above by the fact that *z* must be greater than  in Eq. (4).

This correction procedure is relatively unstable, since it is very sensitive to the arbitrary parameter *α*. All the more so with very low values of *θ*, as on the right of Fig. 6, where the model begins to percolate, i.e., where the runs merge together at a rapidly increasing rate. Nevertheless we see no evidence in the figure that *µ* is much greater than 1, leaving a gene-by-gene model very much a viable candidate alongside the geometric excision model.

## A model for *π*(*r*) in the one-sided model

We are interested in inferring *µ* from the observed distribution of run lengths and the proportion of undeleted terms *θ*. At the outset *θ* = 1. As *t* → ∞, *θ* → 0. We are not, however, interested in *t*, since it is not observable and any time-based inference we can make about *µ* will depend only on run lengths and *θ* in any case. On the other hand, *r*, the number of deletion events per run is an interesting variable since we can assume run length is *rµ* on average, and we can model the evolution of *r* directly in the one-sided model. We consider the distribution *π* as a function of *θ*.

As *π* changes, probability weight is redistributed among several types of run:

1. new runs (*r* = 1) falling completely within an existing run of undeleted terms, not touching the preceding or following run of deleted terms

2. runs that touch, overlap or entirely engulf exactly one previous run of deleted terms with *r* ≥ 1, thus lengthening that run to *r* + 1 events,

3. runs that touch, overlap or engulf, by the skipping process, two previous runs of *r*_1_ and *r*_2_ events respectively, creating a new run of *r*_1_ + *r*_2_ + 1 events, and diminishing the total number of runs by 1, and

4. runs that touch, overlap or engulf, by the skipping process, k > 2 previous runs of of *r*_1_*, …* , *r_k_* events respectively, creating a new run of *r*_1_ + … + *r_k_* + 1 events, and diminishing the total number of runs by *k* – 1. Case 3 above may be considered a special case of this for *k* = 2 and Case 2 for *k* = 1.

The first process, involving a deletion event of length *a* requires a run of undeleted terms of at least *a* + 2. What can we say about runs of undeleted terms? We know that runs of deleted terms alternate with runs of undeleted terms, so that there is one run of the former for each of the latter. The mean length  of the deleted runs should be (1 – *θ*)*/θ* times the mean length  of the undeleted runs:(6)

The distribution *ρ*(*l*) of lengths of the undeleted runs is geometric, since each deletion event creates a randomly placed demarcation between two runs in the sequence consisting of all the remaining terms. The number of terms between two successive such demarcations corresponds to the difference between successive order statistics, and is hence geometrically distributed.

**Figure 7:**
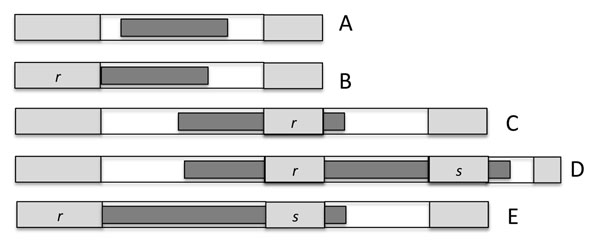
**Deletion model** Types of deletion event affecting less than three pre-existing runs. White area indicates run of undeleted terms. Lightly shaded area indicates run of previously deleted terms. Darker area represents current deletion event. A: creates one new run with *r* = 1. B: lengthens left hand run to *r* + 1 events. C: lengthens right hand run to *r* + 1 events. D and E: merge two runs to create a single run with *r* + *s* + 1 deletion events.

The proportion of terms in runs of length *l* is *lρ*(*l*)/*E_ρ_*, where *E_ρ_* = E_*l*>0_*lρ*(*l*). As depicted in Fig. 7, the probability *p_A_* that a deletion event falls within a run of length *l* without deleting the terms at either end is:(7)

where *j* indexes the starting position of the deletion within the run, and *a* is the number of terms deleted in the event.

The probability *p_B_* that a deletion event touches only the run of deletions on the left of the run of undeleted terms is:(8)

The probability *p_C_* that a deletion event touches or overlaps the run of deletions on the right but does not extend over the entire run of undeleted terms beyond that is:(9)

The probability *p_D_* that a deletion event completely overlaps the run of deletions on the right and touches or overlaps the run of deletions beyond that but does not extend over a further run of undeleted terms:(10)

The probability *p_E_* that a deletion event touches the run of deletions on the left of the run of undeleted terms and touches or overlaps the run of deletions on the right but does not extend over the entire run of undeleted terms beyond that is:(11)

The event A adds one new run with *r* = 1. The events B and C lengthen an existing run from *r* events to *r* + 1. The events D and E join two existing runs of of *r* and *s* events to create a single run of length *r* + *s* + 1. In our initial model, we neglect the merger of three or more runs of deletions. There is no conceptual difficulty in including three or more mergers, but the proliferation of embedded summations leads to computational problems. Thus we should expect the model to be adequate until *θ* gets very small, when mergers of several runs at a time become common.

The last lines of each of (7),(9) and (10) include the collection of terms, significantly cutting down on computing time when these formulae are implemented.

We define the change *δ*(*r*) in the number of runs of deleted terms with *r* = 1, 2,... as:(12)(13)

For *r* > 2,(14)

In an implementation on a finite interval of ℤ, the number of runs of deleted terms will change from some value *R* to *R′*, where:(15)

the distribution of run lengths will also change from *π* to *π*′, where:(16)

where the mean increases accordingly from  to , so that the mean  of the new distribution *ρ′* of run lengths of undeleted terms satisfies:(17)

The new proportion *θ′* of undeleted terms is .

We implement equations (7) to (17) as a recurrence with a step size parameter Λ to control the number of events using the same *p_A_, p_B_, p_C_, p_D_* and *p_E_* and *δ*(·) between successive normalizations, using Λ*δ*(·) instead of *δ*(·) in (15)-(17). The choice of Λ determines the trade-off between computing speed and accuracy.

**Figure 8:**
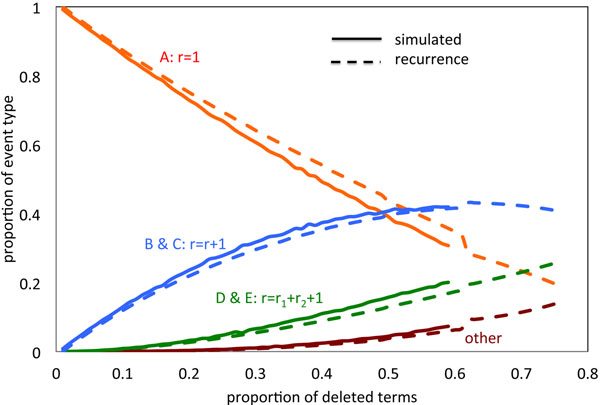
**Comparison of model with simulations** Changes in rates of different event types as calculated by recurrence, compared with simulation results.

Fig. 8 shows the results of our current implementation of our deterministic recurrence for the case *µ* = 2. The results fit simulations of the stochastic model quite well. There are at least two reasons for the observed discrepancies. At the outset, since we used a large step size Λ for the computationally costly recurrence, its trajectory lags behind the simulation, especially with respect to the slower decrease in *p_A_* and slower increase in *p_B_* + *p_C_*. Later discrepancies are partially due to not accounting for the merger of three or more runs. These can be estimated and are summarized as “other ” in the diagram, but the quantities involved are not fed back to the recurrence through (16).

Other possible sources of error might be due to the cutoffs in *x* used for calculations involving γ(*x*) and *ρ*(*x*). However, extensive testing of various cutoff values has indicated such errors to be negligible in our implementation.

## Conclusions

We have developed a model for the fractionation process based on deletion events excising a geometrically-distributed number of contiguous paralogs from one of a pair of homeologous chromosomes. This is extended to the mathematically less tractable case where both homeologs are susceptible to deletion events. The existence of data prompting this model is due to a functional biological constraint against deleting both copies of a duplicate pair of genes.

The mathematical framework we propose should eventually serve for testing the geometric excision hypothesis against alternatives such as gene-by-gene inactivations or imbalanced fractionation, although we have not developed these here.

Simulations of these models indicate the feasibility of estimating the mean *µ* of the deletion event process from observations of the length of runs of single-copy genes and the overall proportion of single-copy genes. Application to real data from an earlier survey of 15 genomes descended from 6 WGD events, however, is hampered by the accumulation of rearrangement events that have obscured most of the runs of single-copy genes. We have proposed a way of correcting for the missing runs, but this remains a rather approximate procedure.

The main outstanding question remains the exact derivation of *π*, the distribution of the number of deletion events contributing to a run of single-copy genes. The simulations are convenient in practice, since they depend on only one parameter *µ* as they evolve over time, but they give little mathematical insight. Our most important advance is a deterministic recurrence for the *π*(*r*) as the proportion *θ* of undeleted genes decreases, albeit for the one-sided model only. This takes into account the appearance of new runs over time, the lengthening of existing runs, as well as the merger of two existing runs with the new deletions to form a single, longer one. This calculation fits the process as simulated rather well and seems promising for further development.

## Competing interests

The authors declare that they have no competing interests.
